# Projecting the effects of land subsidence and sea level rise on storm surge flooding in Coastal North Carolina

**DOI:** 10.1038/s41598-021-01096-7

**Published:** 2021-11-04

**Authors:** Jeremy Johnston, Felicio Cassalho, Tyler Miesse, Celso M. Ferreira

**Affiliations:** 1 Department of Civil, Environmental, and Infrastructure Engineering, George Mason University, 4400 University Dr, Fairfax, VA 22030 USA; 2 Department of Geography and Geoinformation Science, George Mason University, Fairfax, VA USA

**Keywords:** Climate sciences, Environmental social sciences, Hydrology, Natural hazards

## Abstract

Much of the United States Atlantic coastline continues to undergo subsidence due to post glacial settlement and ground water depletion. Combined with eustatic sea level rise (SLR), this contributes to an increased rate of relative SLR. In this work, we utilize the ADvanced CIRCulation model to project storm surges across coastal North Carolina. Recent hurricanes Irene and Matthew are simulated considering SLR and subsidence estimates for 2100. Relative to present day conditions, storm surge susceptible regions increase by 27% (Irene) to 40% (Matthew) due to subsidence. Combined with SLR (+ 74 cm), results suggest more than a doubling of areal flood extent for Irene and more than a three-fold increase for Hurricane Matthew. Considering current regional population distributions, this translates to an increase in at-risk populations of 18% to 61% due to subsidence. Even further, exposed populations are projected to swell relative to Matthew and Irene baseline simulations (8200 and 28,500) by more than 70,000 in all SLR scenarios (79,400 to 133,600). While increases in surge inundation are driven primarily by SLR in the region, there remains a substantial contribution due to vertical land movement. This outlines the importance of exploring spatially variable land movement in surge prediction, independent of SLR.

## Introduction

As much of North America experiences post-glacial rebound following the Pleistocene Epoch, large sections of the east coast of the United States continue to settle. This phenomenon occurs over the mid-latitudes, including coastal New Jersey through South Carolina, as sections of land that were forced upward (forebulge) by glacial loading to the north experience settlement following the loss of ice mass^1,2^. Groundwater extraction has also led to increased subsidence rates along the East Coast from New York to Florida^2–4^. Post glacial settling coupled with continued ground water extraction has resulted in comparatively rapid vertical land movement (VLM) at more than twice the long-term historical rate along large portions of the U.S. Atlantic coastline, exceeding 3 mm/year^2^. While eustatic sea-level rise (SLR) is considered a primary driver of worsening coastal flooding, the specific influences due to changes in land surface elevations, both anthropogenic and due to natural settlement, tend to be overlooked. In the southern Chesapeake Bay region and across North Carolina, these rates are among the fastest in the southeast^5,6^. This contributes to an accelerated increase in storm surge flood risk^7–9^ and can result in tidal flooding, impair drainage systems, and increase reliance on engineered flood protection systems^10–12^. The low elevation gradient and complex coastal features (e.g., barrier islands, sounds, and bays) also suggest this region may respond unpredictably to VLM and SLR^13^.

Rising sea levels remain a significant driver of coastal flooding globally. By 2100, even in low carbon emissions scenarios over 190 million people are expected to be at-risk of tidal flooding alone^14,15^. In coastal North Carolina, anticipated eustatic SLR increases range from around 0.2 m (low) to 3.0 m (extreme), with intermediate projections of eustatic SLR falling between 0.3 m and 1 m by the year 2100^16–19^. As a result, large coastal communities in North Carolina and the southern Chesapeake Bay region (population > 2 million^20^) are at increased flood risk with economic, social, and environmental implications^21–25^. Accumulation of several risk factors is likely to contribute to a notable increase in coastal flooding across the region in the coming decades. These include SLR, sinking ground, growing coastal populations^26,27^, and the potential for more frequent and stronger storms due to warming oceans^28^. Within the last decade alone, high-impact events such as Hurricane Irene and Matthew have directly resulted in a considerable environmental toll^29^, dozens of deaths, and economic losses of an estimated $15.8 billion^30^ and $10 billion^31^, respectively.

Enhanced computational capabilities and numerical circulation models enable the use of computer-based tools for estimating storm surge with a high degree of accuracy^8,32–34^. By perturbing model inputs, numerical modeling approaches have the capability of simulating synthetic storm events. Adjustments to storm track, storm intensity (i.e., central pressure, wind fields), land cover, water levels (i.e., SLR), and underlying digital elevation models (i.e., VLM) can be used to simulate a myriad of scenarios. Coastal North Carolina specifically, provides a well characterized and instrumented candidate region for the use of numerical circulation models. In the region, Peng et al.^35^ estimated over 500 km^2^ of surge flooding from a Category 3 hurricane near the mouth of the Pamlico River. In the neighboring Cape Fear region, the Princeton Ocean Model was effectively used to simulate storm surge dynamics by hindcasting historical storm events^36^. For Hurricane Irene (2011), Loftis et al.^8^ investigated the impacts had it made landfall in 2045, predicting substantial increases in flood extent in the lower Chesapeake Bay. Hydrodynamic modeling along the Gulf Coast has also shown a clear sensitivity of storm surge due to changes in land surface elevations (morphologic and anthropogenic changes), land cover, and SLR^37^. In this region, considerable increases in the extent of storm surge driven flooding are expected by the end of the century, primarily due to sediment re-distribution and SLR^38,39^. These studies, among others, have underlined the usefulness of numerical simulation for surge prediction and risk assessment. However, the unique contributions from eustatic SLR and VLM to future region-wide coastal flooding have not been extensively explored. Specifically, the effect of VLM rates that are not spatially continuous are sparsely considered in the context of storm surge modeling. It remains important to assess regional impacts that may otherwise be missed when considering static VLM or SLR alone. Due to rapid subsidence and an already low-lying land surface, investigation into potentially devastating flooding in North Carolina is increasingly valuable. Hurricanes provide a swift realization of the effects of ongoing regional changes which is in stark contrast to steady and sometimes imperceptible inter-annual variations.

In the following sections, we investigate the effect of land subsidence and SLR in coastal North Carolina with the objective to (1) quantify the increased extent of areas prone to storm surge due to predicted VLM, and (2) to examine the relative contributions of SLR and subsidence to coastal flooding. These efforts have important implications for flood mitigation and planning of coastal resilience by projecting regional vulnerability to storm surge flooding. Similar efforts can be employed globally, especially in regions with extensive histories of land subsidence, to project local contributions to the associated flood risk. Recent works^40^ have highlighted land subsidence as a global challenge, underlining the importance of understanding the unique contributions to flooding from both VLM and eustatic SLR in North Carolina and beyond.

## Methods

### Study region

**Figure 1:**
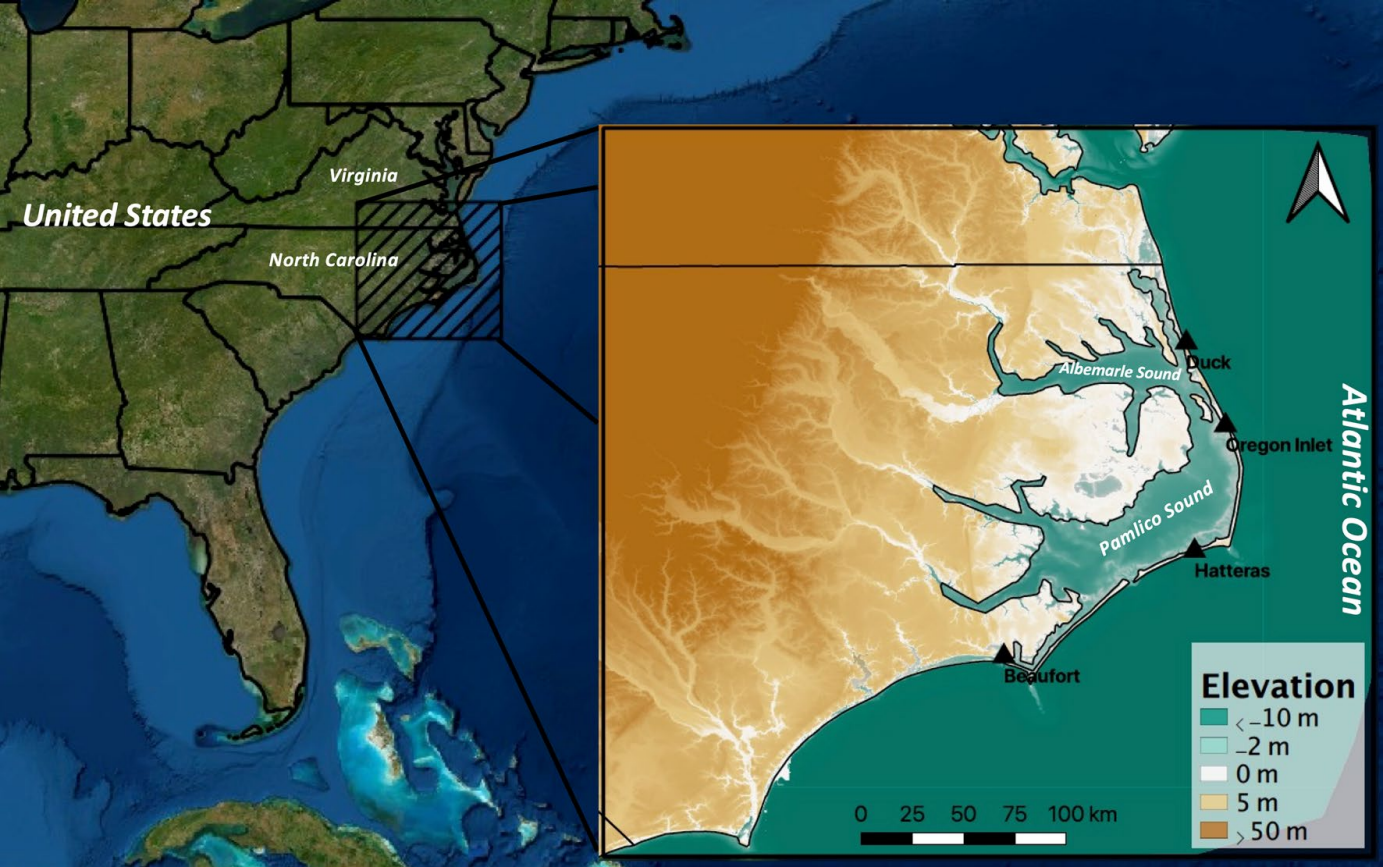
Study region with elevation and bathymetry (CUDEM, Coastal Relief Model, GEBCO, USGS). Water level recording stations indicated. Figure created in QGIS (Version 3.16, https://qgis.org/en/site/forusers/download.html) using ESRI Satellite imagery as basemap.

Coastal North Carolina is home to a unique network of islands, bays, estuaries, and sounds that make up the Albemarle-Pamlico Estuary System (APES, Fig. 1). The region includes Pamlico Sound, a lagoon extending for 80 miles between the North Carolina mainland and the Outer Banks. The area contains the largest saltwater lagoon on the East Coast serving both as an important fishery^41^ and a critical ecosystem^42–44^. The barrier island chain of the Outer Banks separates the APES from the Atlantic Ocean to the east. These islands serve as a breakwater, greatly damping tidal influences and wave action within the APES and connect the sound to the Atlantic Ocean by the Oregon, Hatteras, and Ocracoke Inlets^45^. The region is also prone to storm surge flooding due to the regular occurrence of hurricanes (on average every ~ 2.5 years^46^). This includes recent events such as Irene (2011), Matthew (2016), Florence (2018), and Dorian (2019) as a marked increase in hurricane activity has been shown extending from the early 1990’s through the present day^28,47^. Even further, the region is home to a large tourism and agriculture industry, supporting a considerable population^25^. The complex interplay between these components, coupled with the region wide vulnerability to storm surge flooding^46,48^ make it a challenging yet fascinating case study region for the prediction of coastal flooding.

Figure 1 presents the study region and its topographic and bathymetric profiles. As shown, the region is characterized by shallow waters < 10 m, especially within the APES. Much of the Albemarle-Pamlico Peninsula (A-PP), Outer Banks, and southern shores of the Pamlico Sound also have land surface elevations below 5 m, exposing these areas to storm surge. Coastal wetlands, including both woody and emergent vegetation, provide natural protections against coastal flooding within the region^34^. While also shielded from significant Atlantic influences by the Outer Banks, the region remains susceptible to wind driven storm surges and significant wave action^44,49,50^. Four water level recording stations maintained by the USGS are located at Beaufort, Hatteras, Oregon Inlet, and Duck, NC and provide valuable data inputs for the validation of numerical models (marked in Fig. 1). This data is made available through the NOAA Tides and Currents interactive database (https://tidesandcurrents.noaa.gov/).

### The ADvanced CIRCulation model

The ADvanced CIRCulation (ADCIRC) model is a hydrodynamic circulation model which uses modified shallow-water equations and a triangular mesh to model complex interactions between water bodies and the land surface^51^. The model can accurately represent wind driven currents and waves, considering both the velocity and momentum of fluids to accurately simulate inundation. Many studies have employed ADCIRC to model storm surge and wave action through coupling with the Simulating WAves Nearshore (SWAN^52^) model. Coupled ADCIRC + SWAN has been used to assess potential storm surge in the region surrounding Galveston, TX^33^ and to explore the temporal evolution of changing storm surges across coastal Louisiana^53^. These models have also been successfully employed to study the hydrodynamic response in the Gulf of Maine during notable coastal flooding events^54^. Notably, the North Carolina Forecasting System, developed to provide operational storm surge and wave information to decision makers, also relies on ADCIRC^55^.

For this study, a triangular mesh of more than 800,000 nodes is used to model storm surge over the APES region within ADCIRC. Node elevations are determined from the underlying digital elevation model (DEM), which is derived from the U.S. Coastal Relief Model^56^, the Continuously Updated Digital Elevation Model (CUDEM;^57^), and the General Bathymetric Chart of the Oceans^58^. The effects of vegetation and friction are accounted for using Manning’s n roughness coefficients derived using the U.S. National Landcover Database^59^ using the scheme employed by Liu et al.^60^ and Atkinson et al.^61^. Accounting for land surface features and friction remains critical for accurate water level attenuation in surge modeling, as incorrectly adjusting for these factors has been shown to result in considerable overestimation of inland surge propagation^62^. Atmospheric forcing data are provided in the form of wind fields and surface pressure, forced with hourly data from the European Center for Medium-Range Weather Forecasts (ECMWF ERA5^63^) reanalysis. Further information on the configuration of the coupled ADCIRC + SWAN model is presented in Cassalho et al.^34^.

### Subsidence projections

Land subsidence rates are estimated based on GPS derived vertical change rates presented by Karegar et al.^2^. In this work, they examined displacement trends in GPS elevation data at 216 stations spanning the Atlantic Coast using recent GPS records. The study found the most rapid settlement rates occurring over Mid-Atlantic and southeastern coastal areas (up to − 2.9 mm/year). Slower VLM was observed inland with large parts of the Northeast experiencing uplift. Due to the limited number of GPS stations within the coastal portion of the study region (8), the use of these observations can introduce considerable uncertianty. This is especially true in portions of the region which lie further from observational sites. Even so, the relatively small variability in VLM rates across the region (− 1.89 mm/year to − 2.80 mm/year) support this approach for the estimation of regional averages. Moving forward, implementation of remote sensing approaches combined with GPS observations can prove valuable for tracking VLM rates at finer scales^64–66^.

**Figure 2:**
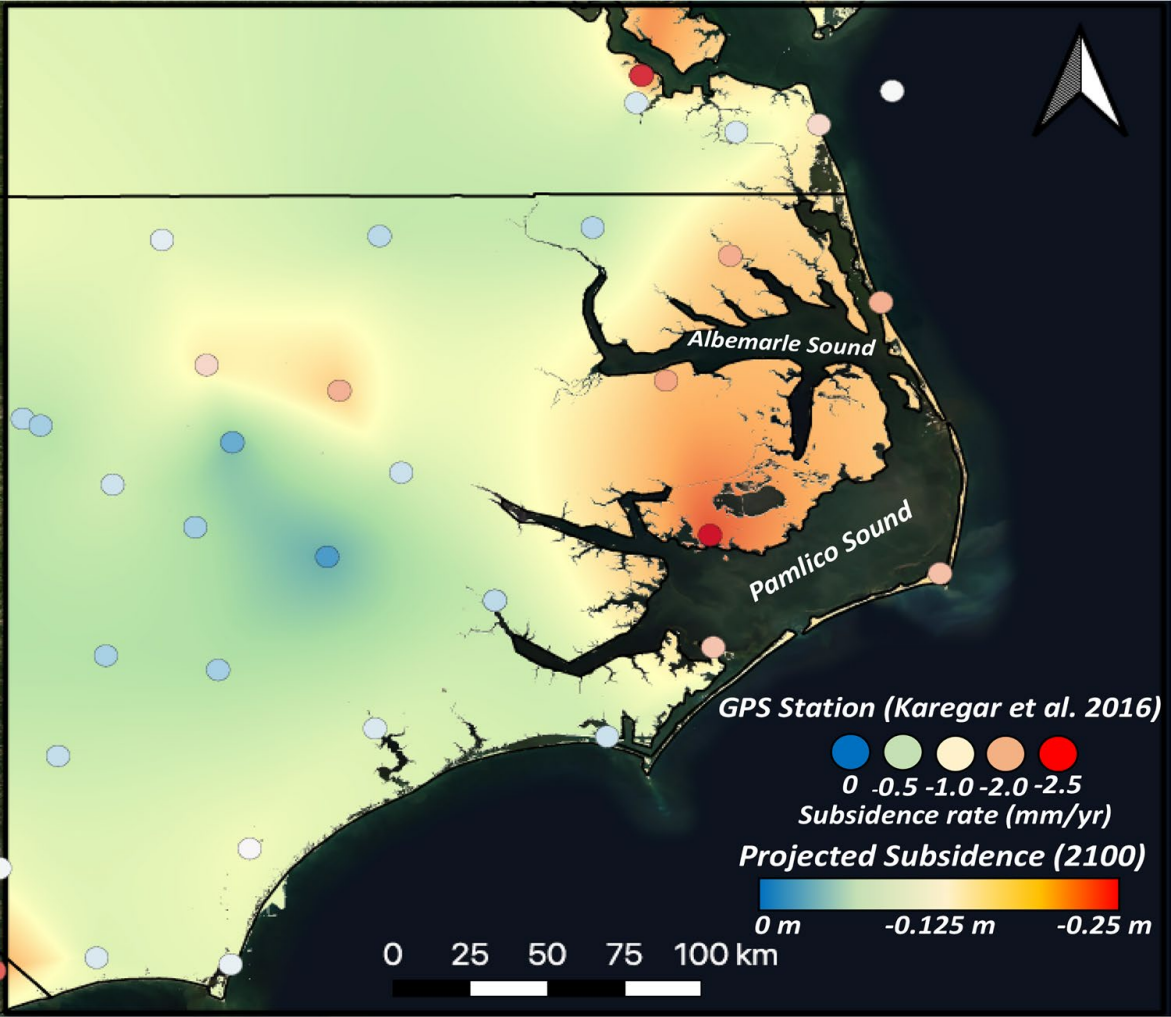
Annual subsidence rates and GPS recording stations, from^2^, and projections of total land surface elevation changes by 2100. Figure created in QGIS (Version 3.16, https://qgis.org/en/site/forusers/download.html) using ESRI Satellite imagery as basemap.

Annual rates derived from GPS stations^2^ were interpolated using the natural neighbor method to create a continuous map of displacement rates across the region (Fig. 2). Using recent DEMs (see section, *The ADvanced CIRCulation Model*) to create the baseline approximation for 2020 surface elevations, projected elevations for year 2100 were calculated using a linear forecast, shown in Eq. (1):

1 $${DEM}_{2100}= {DEM}_{recent}+{S}_{r}(2100-2020)$$

where S_r_ represents the annual rate of VLM at a given cell in the interpolated raster. Vertical displacement projections ranged from − 23 to − 3 cm by 2100, with the highest rates occurring within the A-PP. While annual VLM rates derived from GPS records of limited length (< 20 years) contain uncertainty, by assuming no significant changes in groundwater extraction these rates are expected to remain approximately stable on a decadal timescale^2^. This supports to application of a linear forecast for VLM approximation. Overland mesh nodes in the ADCIRC model were then modified by applying the value from the nearest interpolated cell to the corresponding node elevations. Interpolated VLM rates were not applied to mesh nodes below sea level due to uncertainty presented by sediment redistribution and lack of submerged GPS stations. The majority of coastal regions including the Outer Banks and portions of southern Virginia show estimated subsidence on the order of 7–20 cm by 2100, which is consistent with both USGS and NOAA projections^7,18^.

### Sea level rise projections

As a result of a variety of greenhouse gas emissions scenarios, model uncertainty, and questions regarding the stability of the Antarctic and Greenland ice sheets, there remains a wide range in potential eustatic SLR realizations over the coming decades. Global mean sea levels (GMSL) are expected to increase on the order of 0.25 to 2 m by the year 2100^17–19,67,68^. However, SLR is not regionally constant, with the Western Pacific and Atlantic experiencing more rapid increases^18,19,69^. Along the Atlantic Coast, recent estimates extend from 0.3 to > 2.0 m in the event of high emissions and significant acceleration of glacial melt^16–19^. At sites specific to coastal North Carolina, NOAA projections display a similar range of SLR scenarios (Table 1). Predictions from NOAA^18^ account explicitly for the expected magnitude of VLM and tend to be slightly higher than those estimated by Hall et al.^17^. The high and low end of the presented ranges are unlikely, as one assumes no acceleration in the rate of SLR and others consider rapid glacial melt coupled with ice sheet collapse and increased thermal expansion. Projections in the center of this range thus provide estimates in line with anticipated increases in GMSL and encompass the most likely IPCC scenarios. As such, three SLR approximations of 44, 55, and 74 cm, derived from IPCC projections^19^ were selected for use in ADCIRC simulations. Although the potential exists for SLR above 0.44–0.74 m, especially when using semi-empirical methods and considering high-end projections (Table 1), these values provide a realistic range of global eustatic sea level increases, separate from land movement, captured within the scope of the most probable Representative Concentration Pathways (RCPs). Recent projections from Nerem et al.^70^ predict GMSL increases of 53–77 cm by 2100 (relative to 2005), similar to values used in this work.

**Table 1:** Sea level rise projections for 2100.

Location	Projected vertical land movement (m)	Low (m)	Intermediate (m)	High (m)	Source
Chesapeake Bay Bridge-Tunnel, VA	–	0.48	1.25	2.18	^17^
	0.16–0.20	0.44	1.18	2.59	^18^
Wilmington, NC	–	0.16	0.93	1.86	^17^
	0.03–0.07	0.32	1.02	2.46	^18^
Beaufort, NC	–	0.21	0.96	1.90	^17^
	0.07–0.12	0.36	1.09	2.54	^18^
Oregon Inlet Marina, NC	–	0.23	0.99	1.92	^17^
Southport, NC	–	0.17	0.93	1.86	^17^
Cape Hatteras, NC	0.11–0.17	0.40	1.13	2.36	^18^
Duck Pier, NC	0.13–0.18	0.41	1.16	2.59	^18^

Sweet et al., 2017^18^ consider vertical land movement (VLM) along with changes in global circulation patterns and the Earth’s crust, corresponding to 6 global GMSL scenarios (0.3–2.5 m). These projections rely on GPS observing stations and discretized GMSL projections in line with a range of RCPs. Hall et al., 2016^17^ rely on similar IPCC projections but also incorporate relative SLR rates derived from historical GPS records and land surveys.

### Hurricane simulations

**Figure 3:**
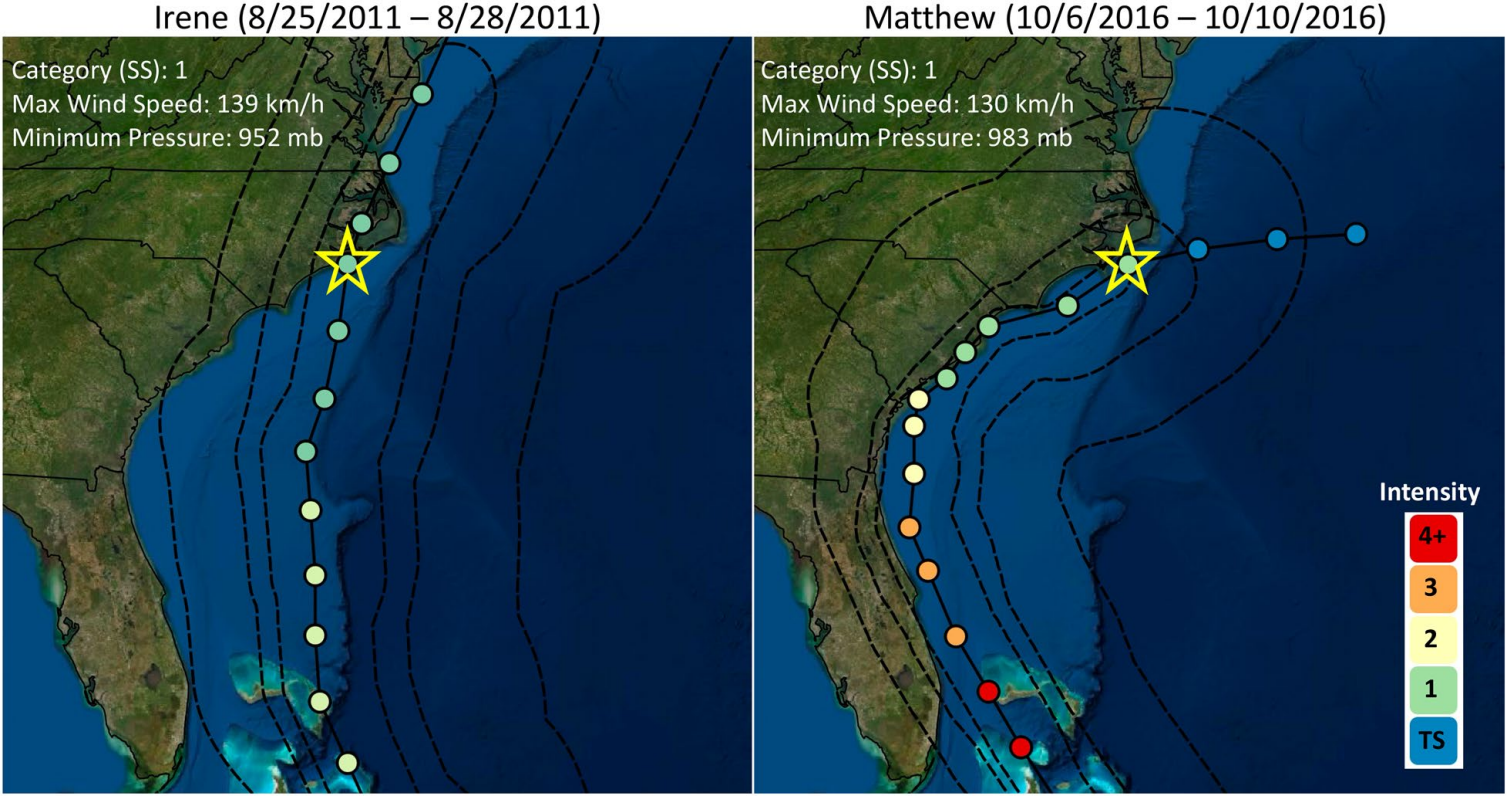
Best track and wind field indicated for hurricanes Irene and Matthew. Wind speed and pressure characteristics recorded for position nearest landfall in North Carolina (indicated by star). Intensity presented based on Saffir-Simpson (SS) scale. Best track information available via the National Hurricane Center, https://www.nhc.noaa.gov/gis/. Figure created in QGIS (Version 3.16, https://qgis.org/en/site/forusers/download.html) using ESRI Satellite imagery as basemap.

Hurricane Irene made landfall in the Outer Banks near Cape Lookout, NC on August 27, 2011, bringing significant storm surge and wave action^30^. While Hurricane Matthew did not directly make landfall in the study region, it approached the coast before veering out to sea on October 10, 2016 (Fig. 3). Both events affected the region with hurricane force winds, with maximum sustained winds on the order of 130–140 km/h (approximately 84 mph). Figure 3 details the storm path and extension of hurricane force and tropical storm force winds from the storm center. While both events crossed the study region with similar strengths, Irene was a direct hit to much of the APES region. Counterclockwise wind circulation pushed sound side water inland and Atlantic waters towards the barrier islands before causing a seaward storm surge towards the sound-side Outer Banks as the system progressed to the northeast.

ADCIRC simulations are forced with ECMWF (ERA5) atmospheric pressure and wind fields. In a recent study by Garzon et al.^71^, ECMWF was shown to most effectively model storm surges in the Chesapeake Bay in a comparison between six unique forcing data sets. In this study, ERA5 based forcing inputs remain unchanged across each set of simulations for both Irene and Matthew. Baseline simulations rely on up-to-date bathymetry and elevation models, land cover, and ERA5 meteorological parameters. Following baseline and validation runs, node elevations and water level datum are adjusted through a static adjustment in the ADCIRC modeling framework to account for subsidence and eustatic SLR. Thus, each storm was modeled with five unique simulations, including current day, subsidence only, and subsidence + SLR (of 44, 55, 74 cm) projections. The resulting node-specific maximum depths and hourly water level outputs were recorded over the duration of study period for both Irene (August 26, 2011–August 29, 2011) and Matthew (October 7, 2016–October 10, 2016).

### Exposed population estimates

Using the Center for International Earth Science Information Network (CIESIN) 1 km resolution population grids, derived from the 2010 Census^20^, we estimated the total populations to be impacted by storm surges based on inundation extent maps derived in this work. Flood extent area, as defined by all 100 m resolution grid cells with positive water surface elevations from ADCIRC outputs, are used to define a binary raster. A raster intersection is performed using GIS software to extract the sum of populations within the intersecting region of the two data grids. As used in this work, population estimates do not consider population change projections within the region to the year 2100, which may increase dramatically^27^. Mismatches in resolution may also result in imperfect estimates as population raster cells with > 50% inundation are included in the intersection. Still, estimates provide general guidance as to the magnitude of expected population displacement due to changes in sea level and land surface elevations.

## Results

### Model validation for Irene and Matthew

**Figure 4:**
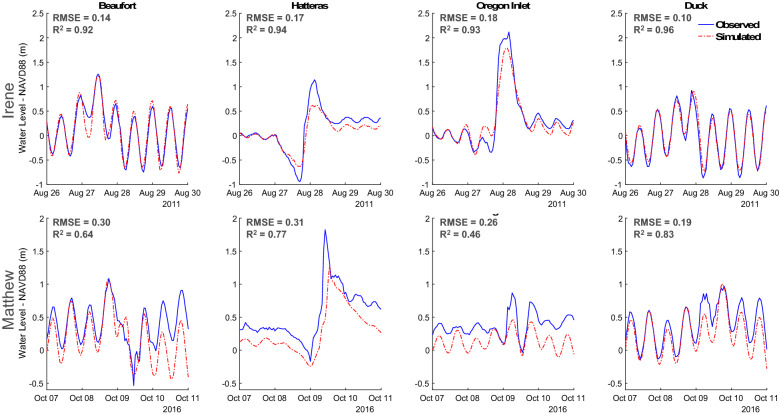
Model validation against NOAA water level gages. RMSE and correlations computed across storm periods, Irene (August 26 0Z–August 30 0Z) and Matthew (October 7 0Z–October 11 0Z).

Here, baseline simulations provide estimates of storm surge extent and water levels due to Hurricane Matthew and Irene as they made landfall. Using USGS gauge stations along the Atlantic coast of North Carolina (Fig. 1), we validated water level observations against simulations to characterize model performance during the period of August 26–30, 2011 (Irene) and October 7–11, 2016 (Matthew, Fig. 4). The resulting baseline simulations provide a reasonable origin point from which to compare future storm surge simulations for the year 2100, as additional flooding due to land subsidence and rising seas can be isolated.

Simulated water levels present strong agreement with observations with an RMSE ranging from 10 to 31 cm and a high average correlation across all sites (> 0.8, Fig. 4). Validation results also suggest acceptable performance in the modeling of storm surge temporal characteristics, as the timing of water level peaks and troughs are shown to be in good agreement both for tidal and storm surge dominant periods. The results suggest sufficient model skill at modeling peak water levels for both events at the Duck and Beaufort observing sites, having errors within 5 cm for both storm events. However, significant underestimation in peak water levels was shown at both Hatteras and Oregon Inlet on the order of 0.3–0.5 m. Both of these recording sites are situated on the sound side of the barrier islands, adjacent to more complex topographic features, highlighting the challenges of accurately modeling water levels in these areas compared to sites situated off the coast. Still, ADCIRC simulations remain accurate, especially for Hurricane Irene, with RMSE’s below 18 cm at all validation sites and very high correlations (R^2^ > 0.92). Simulated water levels for Hurricane Matthew were not as accurate (RMSE 19–31 cm, R^2^ 0.46–0.83). Significant riverine flooding due to excessive rainfall also occurred during Matthew, which was not considered within the model framework. Increases in model underestimation following periods of increased riverine discharge suggest these influences as the primary driver of model underestimation across simulations. Notably contributing to model underestimation of peak water levels. Contributions from rainfall, riverine influences, errors in ERA5 wind forcing, and slight mismatches in initial node water depths all contribute to model uncertainty. Cassalho et al.^34^ provide further model performance and validation statistics using both an assessment of high-water marks (USGS) and modeled wave heights. The study identifies model tendencies to underestimate high-water marks by around 0.5 m. Again, the omission of riverine and rainfall inputs as well as imperfect modeling of friction and landforms (i.e., water attenuation) contribute to this error.

Spatially (see Supplementary Figure S1), flood waters are simulated to inundate over 2100 km^2^ for Hurricane Irene and 1400 km^2^ for Matthew with much of this area being part of the A-PP. This is equivalent to an estimated 1.3 trillion (Irene) and 500 billion (Matthew) liters of water forced overland. For Hurricane Irene, severe flooding occurred in the southern APES with flood depths approaching and exceeding 1 m in areas including Lowland, Stumpy Point, and over much of the central Outer Banks. In contrast, Hurricane Matthew produced more significant flooding towards the northern portion of the APES. This region is also characterized by an extensive area with elevations below 2 m and gentle ground slopes which is shown to contribute to an increased duration of flood water retention following the initial storm surge. Overall, storm surge flooding due to Matthew was generally less severe than that of Irene. Still, both events placed substantial populations in the APES region within the maximum flood extent boundary including around 8000 (Matthew) and 30,000 (Irene) individuals. A summary of impacted populations^20^ by each scenario is included in Table 2. Estimates of flood extent are comparable to maximum inundation depths from hindcasts developed as part of the Coastal Emergency Risks Assessment (CERA) project (https://cera.coastalrisk.live^72^) and NWS modeled flood extents^73^, underestimating maximum overland depths by approximately 0.25 m (Irene) and 0.15 m (Matthew) on average. Thus, model baseline simulations provide an adequate representation of storm impacts in regard to both flood extent and depth and provide a new perspective on the extent of at-risk populations living directly within the storm surge boundary.

**Table 2:** ADCIRC simulation summary statistics for shown APES region.

Simulation	Flood depth (m)			Flooded area			Vulnerable population	
	Mean	Median	Standard deviation	Areal extent (km^2^)	Additional impacted (km^2^)	Flood extent increase (%)	Total	Increase (%)
**Irene**								
Baseline–2011	0.63	0.55	0.37	2196	–	–	28,513	–
*Sub. Only−– 2100*	0.66	0.58	0.37	2795	599	27	33,713	18
*Sub.* + *SLR (44 cm) − 2100*	0.56	0.46	0.42	4104	1908	87	102,523	260
*Sub.* + *SLR (55 cm) − 2100*	0.63	0.54	0.44	4454	2258	103	111,370	291
*Sub.* + *SLR (74 cm) − 2100*	0.74	0.67	0.47	4982	2787	127	133,568	368
**Matthew**								
*Baseline − 2016*	0.39	0.34	0.21	1431	–	–	8,214	–
*Sub. Only − 2100*	0.44	0.41	0.21	2038	607	42	13,278	62
*Sub.* + *SLR (44 cm) − 2100*	0.49	0.45	0.30	4052	2621	183	79,428	867
*Sub.* + *SLR (55 cm) − 2100*	0.56	0.53	0.33	4395	2964	207	93,575	1039
*Sub.* + *SLR (74 cm) − 2100*	0.68	0.67	0.37	4939	3508	245	115,328	1304

Areal overland extent determined as areas with positive water depths over the land surface as defined by the present-day DEM. Vulnerable population statistics derived from CIESIN 2017 datasets (2010 U.S. Census)^20^.

### The relative impacts of subsidence and sea level rise on storm surge

#### Hurricane Irene

**Figure 5:**
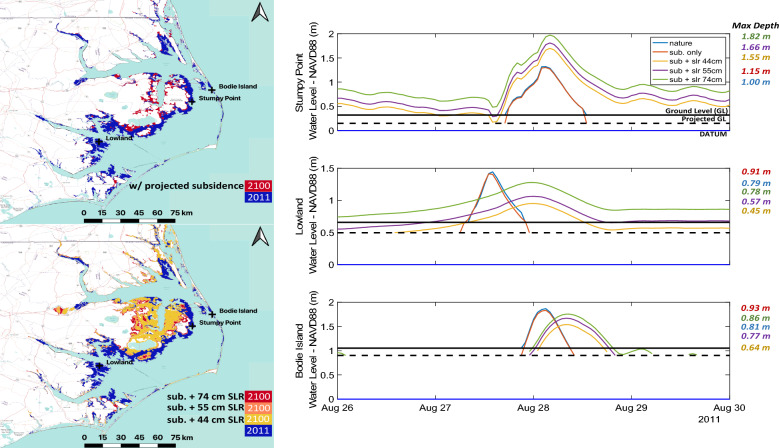
Hurricane Irene flood extent and water level timeseries at indicated focus node locations including Bodie Island, Stumpy Point, and Lowland, NC. Results summarized in Table 2. Figure created in QGIS (Version 3.16, https://qgis.org/en/site/forusers/download.html) using ESRI Transportation and Terrain basemaps.

Figure 5 presents results in terms of spatial extent of maximum flood waters across the APES for Hurricane Irene. Water levels at specified nodes (Bodie Island, Stumpy Point, Lowland) are presented to facilitate the comparison of water level timeseries across simulations. Subsidence alone is shown to increase the extent of flooded area by 27% relative to present day conditions. This increase exposes an additional estimated 5000 individuals to flooding for an event similar to Hurricane Irene in 2100. While notable differences are observed considering only VLM, with the addition of SLR, flooded areas are particularly extensive. Even considering a low SLR scenario (+ 44 cm), by 2100, the areal flood extent produced by a storm similar to Irene is expected to nearly double (+ 87%) placing upwards of 100,000 people at risk in the APES region alone. In the highest modeled scenario (+ 74 cm) the areal extent of flooding nears 5000 km^2^, an increase of 127% compared to baseline simulations. Worsening surges are shown to be focused over the A-PP, which is especially at risk of regular inundation due to its low elevation profile.

Results suggest that approximately half of the A-PP can expect inundation by a storm event equivalent to Hurricane Irene in 2100. In comparing the rate of growth in the flooded area, the no-SLR to + 44 cm scenario produces an increase in flood extent by 60% (1309 km^2^), while the additional flood extent in comparing + 44 cm to + 74 cm of SLR increases by 40% (878 km^2^). This outlines the susceptibility of this region to modest SLR (+ 44 cm) and may suggest non-linearity in surge extent increases. Over the A-PP, Fig. 5 illustrates extensive flood increases relative to baseline simulations (blue) considering 44 cm of SLR and subsidence (dark yellow), while 74 cm of SLR leads to a less profound increase in storm surge susceptible areas (red). Predicted increases in inundation are also prevalent over the southern half of the Outer Banks. Remarkably, at and around Bodie Island, an increase in both the duration and extent of inundation is anticipated, though modeled overland depths are shown to decrease relative to both subsidence only and baseline simulations. This result is counterintuitive, as SLR and land movement are shown to result in reduced overland flood depths at many locations. We hypothesize this is due to a larger inundation area, as water spreads out over the coastal plain thus reducing average storm surge depths. This redistribution of flood waters is shown at both Bodie Island and Lowland nodes. However, locally steeper slopes in close proximity to Stumpy Point Bay result in increased storm surge depths as local topography results in increased surges in this area.

SLR also contributes to a significant shift in storm surge temporal characteristics not seen in subsidence only simulations (Fig. 5). Specifically, SLR simulations predict delayed peak flood timings and increasing flood durations. These results illustrate the complex dynamics between the land surface and storm surge, as increases in maximum flood depths assume a non-linear relationship with SLR. Additionally, many locations are predicted to become part of the tidal basin even in normal conditions (e.g., Lowland and Stumpy Point), which exposes a considerable area to regular tidal flooding.

#### Hurricane Matthew

**Figure 6:**
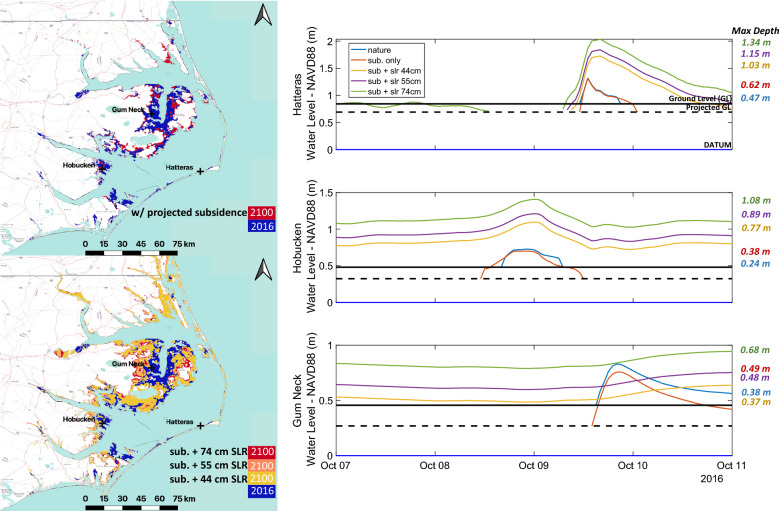
Hurricane Matthew flood extent and water level timeseries at indicated focus node locations including Gum Neck, Hobucken, and Hatteras, NC. Results summarized in Table 2. Figure created in QGIS (Version 3.16, https://qgis.org/en/site/forusers/download.html) using ESRI Transportation and Terrain basemaps.

In Fig. 6 we compare the expected contributions of land subsidence and SLR to increases in flood extent using Hurricane Matthew as the underlying meteorologic forcing. Increases in sea level and decreases in land surface elevations result in flooded areas similar to Irene, but with even more striking increases compared to the baseline simulation. Due to settlement alone, we estimate a more than 40% (+ 607 km^2^) increase in flood extent relative to the baseline (Table 2). Still, SLR is shown to be the primary driver of increases in the regional extent of storm surge. Figure 6 illustrates an increased area exposed to storm surge flooding on the order of 3 × the baseline extent in the most severe scenario (+ 74 cm SLR), expanding by upwards of 240% from 1431 to 4939 km^2^. In this scenario, the areal extent of inundation is almost identical to that of Hurricane Irene considering + 74 cm SLR (4982 km^2^). Highlighting the susceptibility of these areas to storm surge with the expectation of more frequent flooding even considering a variety of storm characteristics (i.e., varied wind fields, approach angles). Results show substantial increases in flood risk in the Outer Banks as well, directly contributing to the considerable rise in affected populations. SLR drives an increase in at-risk populations, from affecting only sparsely populated areas (~ 8000 residents) to more than 115,000 (2100) within APES region alone.

Future simulations also resulted in similar peak water level timing at the Hobucken and Hatteras nodes compared to baseline simulations, with a general delay in maximum flood depth timing on the order of 2 h. In contrast, at Gum Neck which is located at low elevations on the A-PP, we see a considerable delay in peak surge timing. Flood waters are simulated to rise steadily and drain slowly over this area, largely due to topography, as much of the additional simulated flood area is situated below 0.5 m (Fig. 1). Still, the increases in flood depths are not anticipated to be as extreme in the region surrounding the Alligator River such as other non-protected areas (e.g., Hobucken and Hatteras). In Gum Neck, surge depths are shown to remain within 25 cm of baseline projected maximum depths, even when considering SLR considerably exceeding this amount (44–74 cm). This is relative to more exposed locations, where additional wind water interaction and more severe surges are expected. Inundation at Hatteras is shown to increase substantially with the max flood depths rising by nearly 1 m when considering + 74 cm of SLR compared to the baseline. Over both Hatteras and Hobucken nodes, flood duration as a result of Matthew is shown to increase considerably. Our results indicate that much of the low-lying portions of the A-PP and those south of the Pamlico River will be reclaimed by the sound in coming decades due to SLR, becoming uninhabitable as they transition into part of the tidal basin.

## Discussion, significance, and study limitations

SLR is revealed to provide the dominant contribution to increased storm surge flood extents, with increases on the order of 90 to 250% compared to around 30 to 40% in subsidence only simulations (Table 2). In other words, for lower end SLR projections (44 cm), 31% of the increase in flood extent is attributed to VLM (69% SLR) for Irene while this contribution is 23% (77% SLR) for Matthew. For higher-end SLR (74 cm) the VLM contribution becomes 21% and 17% for Irene and Matthew, respectively. Prior literature examining VLM contributions in the Mediterranean^74^, China^32^, and along the U.S. Atlantic coastline^7^ have also illustrated the importance of accounting for VLM when examining region specific SLR implications and drivers. Our results also suggest that both VLM and SLR should be considered when estimating implications of future coastal storm events. Furthermore, as a direct result of these factors, a similar increase in the size of populations exposed to storm surges in many coastal communities is anticipated. In the more severe projections, which consider 74 cm of SLR coupled with land subsidence, over 100,000 additional individuals are likely to be impacted in the region from an event similar to Hurricane Matthew, an increase of over 1300%.

Previous efforts to understand the regional susceptibility to climate change and potential impacts have been made, with comparable findings to that of this work. Over the A-PP prior investigations have suggested that 1 m of SLR could inundate over 40% of the APES, having disproportionate impacts on poor communities^24^. Even further, a global analysis of populations at risk to 0.9 m SLR in 2016 identified over 90,000 residents expected to be at risk in coastal NC by 2100 considering current populations and 165,000 considering population growth rates^27^. These efforts present a range comparable to projections of at-risk populations considering SLR and storm surges here (Table 2). These estimates are based on current populations and would increase if considering expected population growth.

The North Carolina Climate Science Report^75^ identifies increases in heavy precipitation (very likely), significant SLR (virtually certain), increased hurricane intensity (medium confidence), and required changes in associated engineering design standards (very likely) as ongoing or probable effects of climate change. To quantify expected impacts of SLR, Kopp et al.^16^ estimated significant increases in the frequency of severe coastal flooding to occur between 2050 and 2100 in North Carolina, depending on RCPs. As a result, $4 and $17 billion of additional coastal properties are expected to experience regular flooding by 2050 and 2100, respectively^76^. Combining our efforts with findings from such studies suggest that at a decadal timescale, large portions of the region will become unlivable due to more severe and frequent flooding. The results also suggest that even events that provide a glancing blow to the region (Matthew) could have impacts similar to that of a direct hit (Irene) in the future. This is most notable in comparing maximum flood extents in + 74 cm SLR simulations in which the extent of inundated areas converged towards 5000 km^2^. This suggests topographic characteristics in the region that may slow the growth of at-risk areas in the event of additional SLR. Without substantial investment in coastal resilience, the effects of these factors, accelerated by coastal storm events, is expected to reshape the APES along with its communities and ecosystems. Still, the use of regional-scale VLM changes to project surge risk does not explicitly consider the evolution of coastal features (i.e., dune systems) due to sediment deposition and other factors. These changes can be challenging to accurately predict but remain relevant when simulating local-scale surges^37,77^. Still, the projected implications here reiterate those detailed in Poultera et al.^78^, which include additional ecological impacts due to saltwater intrusion, wetland accretion, barrier island section collapse, and loss of waterfront property. Risk reduction policies including investment in engineered protections, relocation programs, and flood insurance should be utilized. The deployment of ecosystem-based approaches such as wetland and dune restoration, beach replenishment, and biogenic reefs are among additional natural solutions with the potential to improve coastal resilience while protecting natural ecosystems^79^.

Multiple considerations and limitations remain important to consider. First, there is uncertainty regarding the maximum water level projections due to known model underestimation of high-water marks. Hydrologic inputs from riverine models and contributions due to rainfall are also largely ignored here, which can be expected to contribute to increased flooding across the APES. Friction estimates using variable Manning’s n estimates also induce additional uncertainty as high-resolution features (e.g., building structures, small landforms) across the region are not explicitly modeled. Other sources of uncertainty include that of bathymetric and ERA5 forcing data inputs, which are also expected to contribute to differences between the modeled and observed water levels (Fig. 4). The lack of available information on the true areal extent of flooding from both Hurricane Irene and Matthew also limits validation of baseline surge estimates. FEMA estimates suggest damages due to Hurricane Matthew were more costly to North Carolina with nearly $400 million in federal assistance allocated^80^ compared to Irene in which approximately $140 million was allocated^81^. In North Carolina alone, Hurricane Matthew damaged or destroyed over 98,000 homes, 19,000 businesses, and a considerable amount of infrastructure (e.g., roads, dams) suggesting underestimation of affected populations by this study^82^. This may be due in part to the use of outdated population statistics (2010), however, requests for federal aid also incorporate damages due to high winds and riverine flooding statewide. Therefore, the proportion of damages contributed by storm surge are nearly impossible to determine, complicating model validation. More severe surges were observed during Irene compared to Hurricane Matthew, even as total damages remained significantly lower. This suggests that the relative contribution of storm surge to total event damages was considerably larger in the case of Irene. Uncertainty also exists in VLM projections as these are based off of a linear forecast from a relatively sparse network of historical GPS sites^2^. Even so, projected VLM biases are on the order of a few centimeters as historical subsidence rates do not tend to change significantly over a timescale of 80 years, except in cases of rapid groundwater depletion. Even further, small shifts in land surface elevations are not shown to be the primary driver of increasing storm surges within the region. While the use of GPS sites for projection of VLM is valuable at the point scale and for the projection of regional average subsidence rates, this method is not capable of capturing VLM contributions at high spatial resolutions. These limitations should be considered when interpreting results. Moving forward, the use of remote sensing approaches (i.e., InSAR) is expected to provide valuable inputs for producing improved local-scale projections^64–66^. Finally, SLR projections rely on IPCC guidance and contain a considerable amount of uncertainty. The range used here (44–74 cm) encompasses a few possible scenarios of GMSL changes by 2100 from the IPCC’s Fifth Assessment Report^19^. Semi-empirical approaches^83^ suggest the potential for even more significant mean sea level rises (up to 1.4 m by 2100, relative to 1990) but are not backed by scientific consensus. Different emissions pathways and stability of near-polar ice sheets will largely determine the rate at which SLR is realized. Compared to region specific estimates of SLR included in Table 1, the range of relative SLR increases considered here (47–97 cm, eustatic SLR + VLM) remain on the low to intermediate range of relative SLR guidance. This suggests potential for more severe surges than modeled in the coming decades. Similar efforts to this work should be employed at regional scales globally, to accurately project storm surges and the major contributors to flooding (SLR or VLM). The addition of synthetic hurricane/typhoon events may also be valuable in determining potential coastal flood effects from stronger storms in both the Atlantic and Pacific basins and the local impacts of sea level changes and subsidence.

## Conclusions

Numerical modeling presents an effective method for estimating and projecting storm surges and their impacts into the future. In this paper, we utilize ADCIRC to examine additional storm surge of two recent hurricane events (Irene and Matthew) considering both land subsidence and eustatic sea level rise across the Albemarle-Pamlico Estuary System. Using estimates for 2100, we examine the areal extent, at-risk populations, as well as flood timing and depth changes associated with subsidence and SLR. The results show that while vertical land movement contributes to a significant increase in the areal extent exposed to storm surge flooding (+ 27% and + 40%), eustatic SLR is expected to be the primary driver of worsening storm surge impacts for coastal North Carolina. For Irene, the combination of land settlement and low-end IPCC SLR projections of 44 cm, resulted in an increase in the storm surge-prone area from 2196 km^2^ to around 4100 km^2^, an increase of 87%. Considering higher-end SLR projections of 74 cm, this increase is even more pronounced, expanding from around 2200 km^2^ to nearly 5000 km^2^, an increase of 127%. In the case of Matthew, simulations showed an even more devastating increase in flooded areas, with a nearly 250% increase in flood extent in the high-end scenario (1431 to 4939 km^2^) and more than a 180% increase even considering low-end SLR estimates (1431 to 4052 km^2^). The relative contribution from VLM to increased flood extents ranged from 17 to 31% across simulations, with lower contributions occurring as a result of higher-end SLR scenarios. Results also suggest an increase in affected populations of approximately 74,000 to 105,000 (260 to 370%, Irene) and 71,000 to 107,000 (870 to 1300%, Matthew) when comparing 2100 scenarios to baseline simulations in coastal North Carolina. While a small portion of this increase can be attributed to VLM alone, the modeled increases in at-risk populations of 18% (Irene) and 62% (Matthew) are considerable.

With the duration of storm surge inundation also shown to increase, additional erosion and property damage will be increasingly likely. Interestingly, the results also suggest that while flood depths are anticipated to increase, this does not occur equivalent to the amount of SLR. Model results show much of the additional water is expected to spread out over a considerably larger area, skewing the distribution of flood depths lower and resulting in a relationship between increasing storm surge depths and SLR that is non-linear and location dependent. This is in contrast to the projected increase in surge depth due to land subsidence alone which displays a more predictable linear rate of increase. Most importantly, our results illustrate that by 2100 markedly more severe storm surges can be expected from hurricanes of equivalent intensity today, even considering Category 1 events. The specific contribution due to VLM should also be explicitly considered to accurately project storm surge risk. As a result, we expect worsening flood impacts in terms of more frequent and damaging outcomes to populations, coastal ecosystems, and infrastructure. In response, efforts should be employed such as ecosystem-based approaches, engineered flood protections, relocation, and land use planning to limit losses in the coming decades. These results also suggest that community resilience planning and flood insurance mapping efforts should account for the contributions of land subsidence along with eustatic SLR in the APES and beyond, considerations not currently made in federal flood plain assessments nationwide.

## Supplementary Information

Supplementary Legends.

Supplementary Figure S1.

Supplementary Information.

## Data Availability

Derived subsidence maps and maximum flood extent raster files from each of the simulations are available as GeoTIFFs (contact: jjohns60@gmu.edu). Raw simulation outputs as netCDF (.nc) files are available upon request (contact: fcassalh@gmu.edu). ECMWF ERA5 forcing inputs are available via Copernicus at https://cds.climate.copernicus.eu/cdsapp#!/dataset/reanalysis-era5-single-levels?tab=form. The GIS package used in this work is open access (QGIS, https://qgis.org/en/site/) and analysis protocols rely on this (or similar) software.
