# Supplementary material for: Projecting the effects of land subsidence and sea level rise on storm surge flooding in Coastal North Carolina
Source: Sci Rep. 2021 Nov 4;11:21679. doi: 10.1038/s41598-021-01096-7 (PMC8568897; doi:10.1038/s41598-021-01096-7)
Supplement: Supplementary file 1 — Supplementary Legends. [file 41598_2021_1096_MOESM1_ESM.docx]

Supplementary Fig. – Baseline simulation results. Overland flood depth and water level timeseries at indicated node locations for Hurricane Irene (top) and Matthew (bottom). Results summarized in Table 2. Figure created in QGIS (Version 3.16, <https://qgis.org/en/site/forusers/download.html>) using ESRI Transportation and Terrain basemaps
