# Supplementary figures and images for: Projecting the effects of land subsidence and sea level rise on storm surge flooding in Coastal North Carolina
Source: Sci Rep. 2021 Nov 4;11:21679. doi: 10.1038/s41598-021-01096-7 (PMC8568897; doi:10.1038/s41598-021-01096-7)

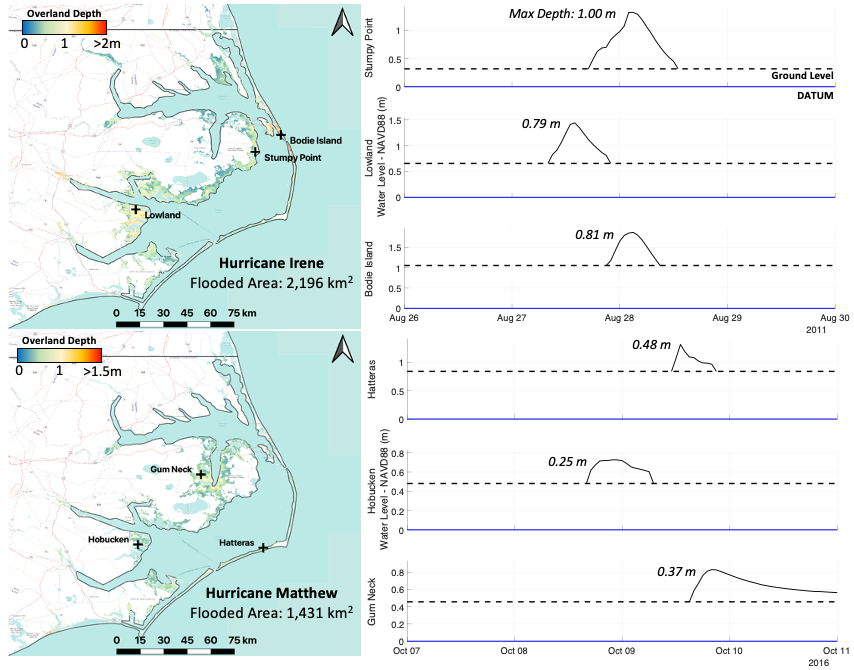

Supplement: Supplementary file 2 — Supplementary Figure S1. [file 41598_2021_1096_MOESM2_ESM.tiff]
