# Supplementary material for: Projecting the effects of land subsidence and sea level rise on storm surge flooding in Coastal North Carolina
Source: Sci Rep. 2021 Nov 4;11:21679. doi: 10.1038/s41598-021-01096-7 (PMC8568897; doi:10.1038/s41598-021-01096-7)
Supplement: Supplementary file 3 — Supplementary Information. [file 41598_2021_1096_MOESM3_ESM.docx]

**Supplementary Information and Supporting Data**

For Manuscript: *Projecting the Effects of Land Subsidence and Sea Level Rise on Storm Surge Flooding in Coastal North Carolina*

Subsidence rates (via Karegar et al. 2016, Supporting Information): <https://agupubs.onlinelibrary.wiley.com/doi/full/10.1002/2016GL068015>

Modeling supporting information (via Cassalho et al. 2021, Text & Supplementary Information):

<https://link.springer.com/article/10.1007/s13157-021-01443-4#Sec15>

Population Data (via Socioeconomic Data and Applications Center (NASA)): <https://sedac.ciesin.columbia.edu/data/set/usgrid-summary-file1-2010/data-download>

Derived Maximum Flood Extent Maps (via Google Drive, GeoTIFF format): <https://drive.google.com/drive/folders/1mJ4y_C0sL1Eb0HwzKiiMuXxT8VVIL18K?usp=sharing>

Raw ADCIRC Model Output Files (.nc): available upon request to the George Mason Flood Hazards Research Lab – via [jjohns60@gmu.edu](mailto:jjohns60@gmu.edu) or [fcassalh@gmu.edu](mailto:fcassalh@gmu.edu)
